# Gas Density Within the Pancreas: A Case of Emphysematous Pancreatitis

**DOI:** 10.5334/jbsr.3331

**Published:** 2023-11-24

**Authors:** Laurens Hutsebaut, Elyn Van Snick, Bart Claikens

**Affiliations:** 1 Radiology Department, AZ Oostende, Oostende, BE; 2 Vrije Universiteit Brussel, BE

**Keywords:** pancreatitis, emphysematous pancreatitis, necrotizing infection, gas density, computed tomography

## Abstract

**Teaching Point:** Computed tomography is the imaging modality of choice to detect the presence of gas within or around the pancreas in emphysematous pancreatitis.

## Case History

A 65-year-old man with a medical history of type 2 diabetes, cardiovascular disease, and primary hyperparathyroidism presented at the emergency department with acute onset of continuous epigastric pain. The abdomen was diffusely tender on clinical examination. Blood results showed a slightly inflammatory features (C-reactive protein (CRP) 16.5 mg/L) with elevated white blood cell count (WBC) of 15 000/mm^3^. Liver tests were elevated with a total bilirubin of 1.47 mg/dL. Lipaemia was strongly elevated, more than three times the normal value (12820 U/L). There was also a hypercalcemia (2.83 mg/dL).

**Figure 1:**
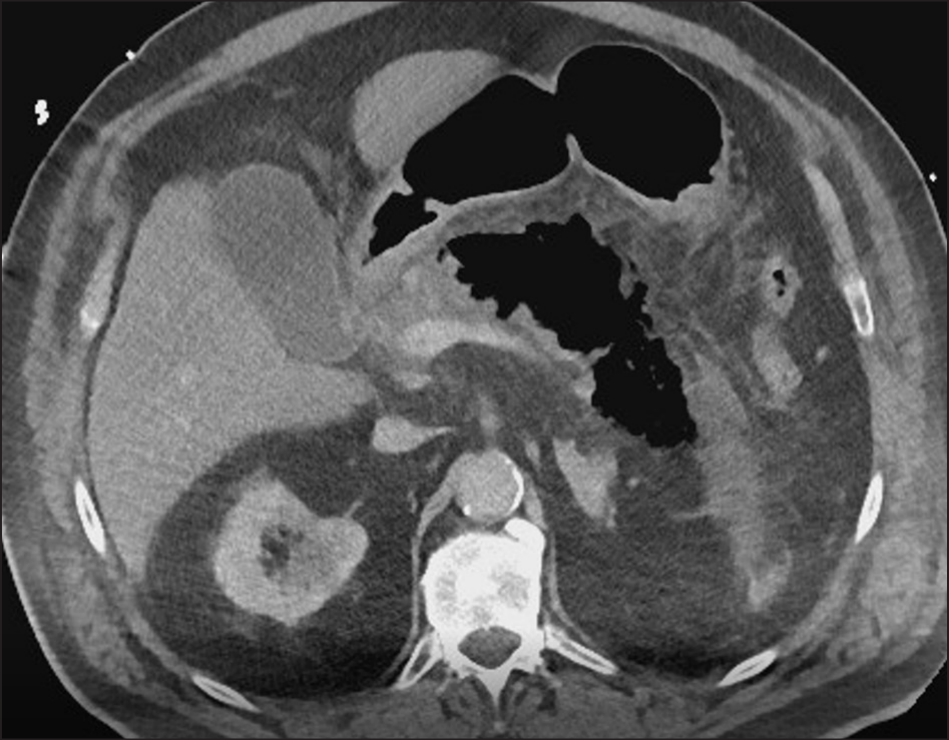


**Figure 2:**
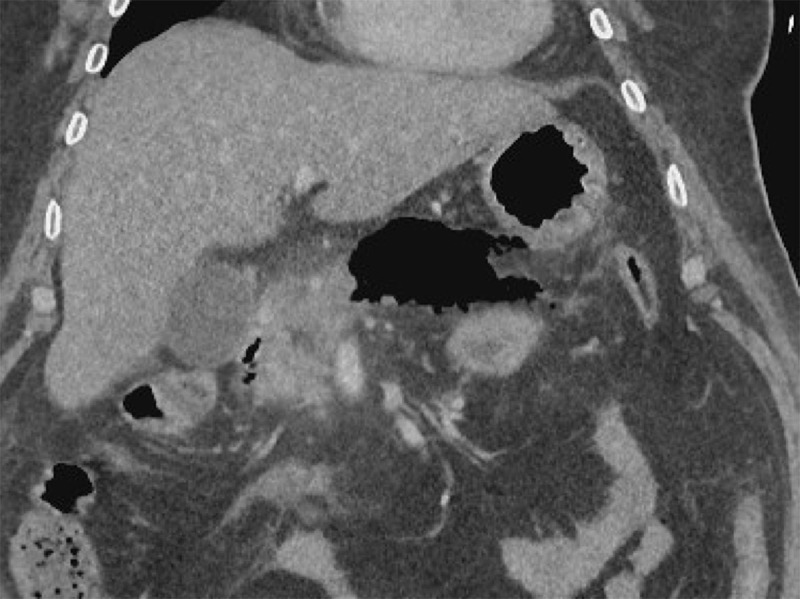


**Figure 3:**
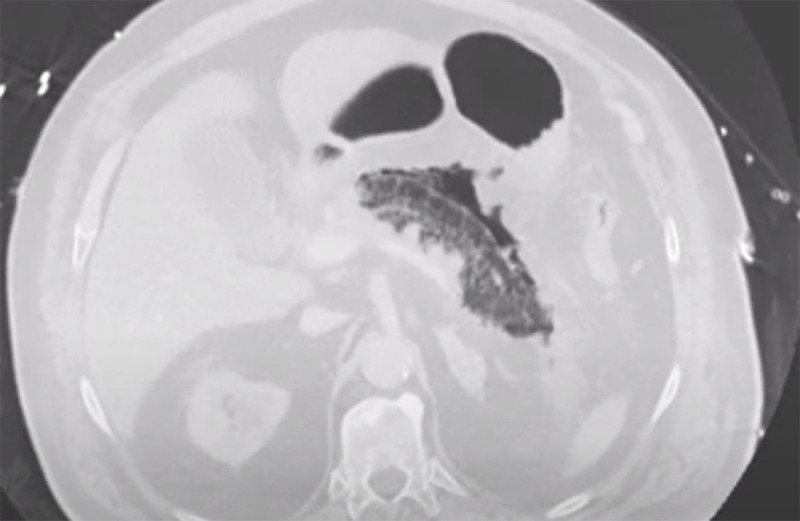


An abdominal ultrasound study was performed, which showed multiple lithiasis and sludge in the gallbladder. The pancreas could not be visualised due to air interposition. The clinical diagnosis of pancreatitis was made, either from biliary origin or due to hypercalcemia, and the patient was admitted for hospital stay for supportive therapy. A computed tomography (CT) scan was performed 48 hours later to evaluate the local status. A large gas collection in and around the body and the tail of the pancreas was observed, along with peripancreatic fat stranding and some fluid (axial scan Figure 1 and coronal reformatting Figure 2). The findings are consistent with emphysematous pancreatitis. Broad window settings nicely depict the presence of gas in and around the pancreatic parenchyma (Figure 3).

## Comments

Emphysematous pancreatitis is a rare and life-threatening complication of acute necrotizing pancreatitis. It is a superinfection of necrotic pancreatic tissue with gas-forming organisms and is characterized by the presence of gas collections within or around the pancreas. Escherichia Coli (E. Coli) is the most frequently identified causative organism. It mainly occurs in immunocompromised patients. Patients with diabetes are at high risk.

CT scan is the imaging modality of choice to diagnose emphysematous pancreatitis in clinically and biologically suspected patients, because of its high sensitivity for the detection of gas density within the pancreatic tissue. The diagnosis is confirmed by isolating the causative organism from peripancreatic aspiration fluid.

Treatment consists of broad-spectrum antibiotics, supportive therapy, and if relevant and feasible, percutaneous drainage of the necrotic fluid collection, and in some cases surgical debridement.

The differential diagnosis includes air that is present after recent surgery or a procedure such as an endoscopic retrograde cholangiopancreaticography (ERCP) or with air originating from an entero-pancreatic fistula [1].

## Competing Interests

The authors have no competing interests to declare.
